# *CopperNostics*—Here We Are Now, Entertain Us!

**DOI:** 10.3390/ph19020321

**Published:** 2026-02-15

**Authors:** Santiago Andrés Brühlmann, Martin Walther, Klaus Kopka, Martin Kreller, Oliver C. Kiss

**Affiliations:** 1 Institute of Radiopharmaceutical Cancer Research, Helmholtz-Zentrum Dresden-Rossendorf (HZDR), Bautzner Landstrasse 400, 01328 Dresden, Germany; s.bruehlmann@hzdr.de (S.A.B.); m.walther@hzdr.de (M.W.); k.kopka@hzdr.de (K.K.); m.kreller@hzdr.de (M.K.); 2 Faculty of Chemistry and Food Chemistry, School of Science, TUD Dresden University of Technology, Mommsenstrasse 4, 01062 Dresden, Germany

**Keywords:** copper radioisotopes, radionuclides, nuclear medicine, PET, SPECT, endoradiotherapy, theranostics, *CopperNostics*, clinical trial

## Abstract

Diagnosis and endoradiotherapy using copper radioisotopes—defined as Theranostics or, more specifically, *CopperNostics*—have the potential to play a prominent role in modern precision medicine, as demonstrated by the FDA approval of [^64^Cu]Cu-DOTA-TATE (Detectnet). In this review we highlight current developments in the production, radiochemical purification, quality control, availability, logistics, and regulatory hurdles of the most relevant copper radioisotopes, ^60^Cu, ^61^Cu, ^62^Cu, ^64^Cu, and ^67^Cu, for nuclear medicine. Radiopharmaceuticals based on their application in registered clinical trials, either as molecular imaging agents, companion diagnostics or therapeutic agents, are also presented addressing unmet medical needs.

## 1. Introduction

Nuclear medicine and radiopharmaceutical sciences play a key role in modern precision medicine, enabling non-invasive diagnosis, therapy, and treatment monitoring [1]. By combining highly specific targeting vectors, so-called tracer molecules, with radioactive nuclides, radiopharmaceuticals allow, on the one hand, the visualization of biological processes in vivo and, on the other, the selective delivery of cytotoxic radiation to diseased tissue [2].

In addition, their biochemical and pharmacokinetic properties can be fine-tuned through structural modifications, enabling optimized biodistribution, clearance, and target retention. Together, these advances support patient stratification and individualized treatment planning [3]. A major development in recent years has been the emergence of radionuclide theranostics, in which diagnostic and therapeutic applications are linked through chemically identical or closely related molecular constructs [4,5].

Radionuclides are unstable isotopes of an element that decay by emitting ionizing radiation, which can be exploited for either diagnostic or therapeutic purposes depending on its physical characteristics. Therapeutic efficacy is most commonly achieved using α-particles, β^−^ particles, conversion electrons, or Meitner–Auger electrons [6]. These particles differ not only in mass, but also in energy and thus in tissue penetration and linear energy transfer.

In contrast, γ-ray and β^+^ emitters are employed for diagnostic imaging through single-photon emission computed tomography (SPECT) or positron emission tomography (PET), respectively, enabling qualitative/quantitative assessment of tracer biodistribution, i.e., target engagement [2]. Parallel to these technological advances, an increasing number of disease-associated molecular targets are being identified and exploited for radiopharmaceutical applications, alongside a rapidly expanding repertoire of radionuclides with diverse physical and chemical properties [4,7].

In some cases, a single radionuclide emits multiple types of radiation and can therefore intrinsically fulfill a theranostic role, as exemplified by ^177^Lu, which combines β^−^ emission for therapy with γ emission suitable for imaging and dosimetric evaluation [8,9]. Such dual-function radionuclides underscore the conceptual and practical advantages of matched diagnostic–therapeutic approaches and have stimulated sustained interest in radionuclide families that offer complementary decay characteristics within a coherent chemical framework—such as the copper radioisotopes discussed in this review and fine-tuned by our group as portmanteau *CopperNostics*, which have the potential for a paradigm shift, particularly, for the true theranostic pair ^64^Cu/^67^Cu [5,10].

## 2. Copper Radionuclides for Imaging and Therapy

**Figure 1:**
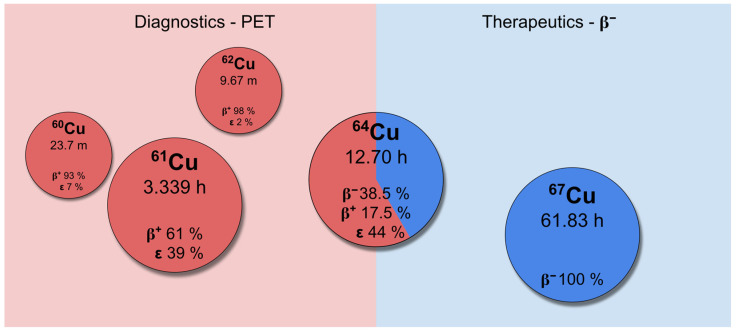
Summary of copper radioisotopes relevant for nuclear medicine purposes.

Copper offers a uniquely versatile radionuclide portfolio, including three positron emitters (^60^Cu, ^61^Cu and ^62^Cu), one ambidirectional β^+^/β^−^ emitter (^64^Cu) and a ‘pure’ β^−^ emitter (^67^Cu). These distinct decay characteristics of copper radionuclides can be exploited in the field of nuclear medicine. Indeed, such characteristics can be utilized for diagnostic imaging, therapeutic interventions and efficient follow-up procedures, enhancing the so-called theranostic approach [4,11,12]. The utilization of neutron-deficient copper radioisotopes, such as ^60^Cu, ^61^Cu, and ^62^Cu, in PET imaging enables the precise localization of lesions with an accuracy of up to a few millimeters [13]. Conversely, ^67^Cu, as a low-energy β^−^-emitter, has potential for therapeutic applications. Its biodistribution can be readily monitored using SPECT imaging, a process facilitated by its γ co-emission [14,15]. Last but not least, the ^64^Cu isotope has been identified as a potential candidate for both applications due to its dual-mode decay pathway [16,17]. A summary of these copper radioisotopes is illustrated in Figure 1 (properties from [18]).

While early investigations focused on ^60^Cu and ^62^Cu, their short half-lives restricted clinical utility, shifting attention toward the longer-lived isotopes: ^61^Cu, ^64^Cu, and ^67^Cu [17,19]. This subset is particularly attractive, as it combines two diagnostic radionuclides (^61^Cu and ^64^Cu) with a therapeutic counterpart (^67^Cu), offering the possibility of a “true theranostic matched pair” [4,19,20,21]. Moreover, radiocopper stands out among theranostic radionuclide families (e.g., ^43/44^Sc/^47^Sc, ^152/155^Tb/^161^Tb [22,23,24]) by uniquely providing two positron emitters with low β^+^ energies and complementary physical half-lives, an advantage that enhances both image quality and clinical flexibility. In addition, this characteristic allows tailoring to the pharmacokinetics of different tracer molecules, improving alignment between biological and physical half-lives. In particular, the physical properties and typical applications of each copper radioisotope are presented in Table 1 (properties from [18]).

**Table 1:** Physical properties of copper radionuclides of interest for nuclear medicine.

Radionuclide	Half-Life	E_β+,mean_/keV (Intensity/%)	E_β−,mean_/keV (Intensity/%)	Eγ/keV (Intensity/%)	Reported Applications
^60^Cu	23.7 m	970 (93)	-	1333 (88) 1792 (45.4) 826.4 (21.7) 3124 (4.8) 1862 (4.8) i.a.	Perfusion [13] Hypoxia [25]
^61^Cu	3.34 h	500 (61)	-	282.9 (12.7) 656.0 (10.4) 67.41 (4.0) 1185 (3.6) i.a.	Hypoxia [25] PSMA [26] Peptide [27]
^62^Cu	9.67 m	1320 (97.8)	-	1173 (0.34) i.a.	Hypoxia [25] Perfusion [28]
^64^Cu	12.7 h	278 (17.5)	191 (38.5)	1346 (0.47)	Perfusion [29] Hypoxia [25] PSMA [30] Peptide [31] Antibody [32] FAP [33]
^67^Cu	61.8 h	-	141 (100)	184.5 (48.7) 93.3 (16.1) 91.3 (7.9) i.a.	PSMA [34] Peptide [35] Antibody [36]

As summarized in Table 1, the listed positron emitters differ substantially in both positron energy and physical half-life, parameters that have direct implications for PET imaging. Lower positron energies translate into shorter positron ranges in tissue and, therefore, improved spatial resolution, whereas higher energies impact negatively in image quality. On the other hand, longer half-lives may enable centralized production and distribution, while shorter half-lives require on-site production. In addition, half-life differences influence the compatibility of each radionuclide with tracer pharmacokinetics and clinical workflows.

Within this framework, some groups favor ^61^Cu for diagnostic imaging, citing its shorter half-life and higher β^+^ branching ratio, while others prefer ^64^Cu for its longer half-life and lower positron energy [37,38]. Ultimately, the choice is likely to depend on the pharmacokinetic profile of the radiotracer—whether rapid uptake and clearance favor ^61^Cu, or slower kinetics are better matched to ^64^Cu.

### 2.1. Radionuclide Production

Copper radioisotopes have been typically produced from cobalt, nickel, copper or zinc targets, each route presenting distinct advantages and inherent limitations. Depending on the target material, production strategies span photonuclear reactions, neutron irradiation, and charged particle-induced reactions, each offering distinct trade-offs in terms of yield, radionuclidic purity, and facility accessibility. Production from cobalt remains the least common, relying on the use of α-particles or ^3^He ions [39,40]. Nickel targets often afford favorable yields and easy separation chemistry but usually rely on expensive enriched isotopic compositions [41,42]. Moreover, direct production from stable copper is generally unsuitable for many radiopharmaceutical applications due to the resulting low molar activity [41,43]. Last but not least, the chemistry of copper separation from zinc has been particularly challenging, requiring more elaborate purification strategies to achieve clinical-grade product in addition to, in some cases, required enriched starting material [15,44].

After initial investments in materials and equipment, the costs for ^64^Cu- and ^61^Cu-radiopharmaceuticals can be comparable to those of established ^18^F-radiopharmaceuticals. As with ^18^F, the main cost factors are laboratory operating costs, energy requirements, and personnel costs. Since these predominant costs can vary significantly from country to country, a quantitative price indication on an international scale is not very useful. Currently, production takes place predominantly in scientific institutions on an experimental scale and is, therefore, not optimized from an economic point of view, because in the scientific field, economic efficiency is not the primary focus, but rather the collection, evaluation, and publication of scientific findings. These findings, however, are a prerequisite for establishing cost-efficient production on an industrial scale.

Taken together, these considerations underscore that the choice of target material, nuclear reaction, and separation chemistry (i.e., radiochemical processing and target material recovery) must be carefully balanced against practical aspects such as molar activity, radionuclide purity, scalability, accessibility to the production facility, and distribution logistics. This complex interplay explains why, despite decades of research, production of copper radioisotopes continues to evolve and remains a highly dynamic field [15,38,41].

#### 2.1.1. Copper-60 and Copper-62

The short-lived radionuclides ^60^Cu and ^62^Cu can be efficiently produced with high yields via the ^60^Ni(p,n)^60^Cu and ^62^Ni(p,n)^62^Cu nuclear reactions, respectively. Although these routes require enriched nickel targets, the relatively high cross sections of the (p,n) reactions enable substantial yields, with activities in the 100 GBq range achievable after one-hour irradiations [45,46]. Alternative production has been investigated exploiting mono-isotopic ^59^Co as the target through nuclear reactions such as ^59^Co(α,3n)^60^Cu and ^59^Co(^3^He,2n)^60^Cu for ^60^Cu, as well as ^59^Co(α,n)^62^Cu for ^62^Cu [39,40]. An alternative approach involves generator-based production of ^62^Cu from its parent radionuclide ^62^Zn [28,47]. While this method can simplify availability, the parent isotope’s half-life (9.2 h) is considerably shorter than that of other copper radioisotopes, limiting practical distribution.

Because of their short half-lives, ^60^Cu and ^62^Cu were particularly attractive for early investigations of radiotracers with rapid pharmacokinetics. In this context, they were primarily evaluated for labeling perfusion and hypoxia tracers such as [^60/62^Cu]Cu(II)-pyruvaldehyde bis(N^4^-methylthiosemicarbazone) (PTSM) and [^60/62^Cu]Cu(II)-diacetyl-bis(N^4^-methylthiosemicarbazone) (ATSM) [25,28,48,49,50]. However, their clinical translation has remained limited, since their use requires on-site production. Moreover, the relatively high positron energies of both radioisotopes—and, in the case of ^60^Cu, the emission of intense γ-rays—adversely affect PET image quality (see Table 1) [13].

Although advances in PET technology, including ultra-fast imaging systems, may improve sensitivity, the very short half-lives of ^60^Cu and ^62^Cu remain a major practical limitation. The growing emphasis on centralized radionuclide production and distribution strongly favors longer-lived isotopes, as reliable logistics and broad clinical implementation are difficult to achieve with radionuclides requiring on-site cyclotron access. Consequently, research and clinical interest have progressively shifted toward the longer-lived positron emitters ^61^Cu and ^64^Cu.

#### 2.1.2. Copper-61

**Figure 2:**
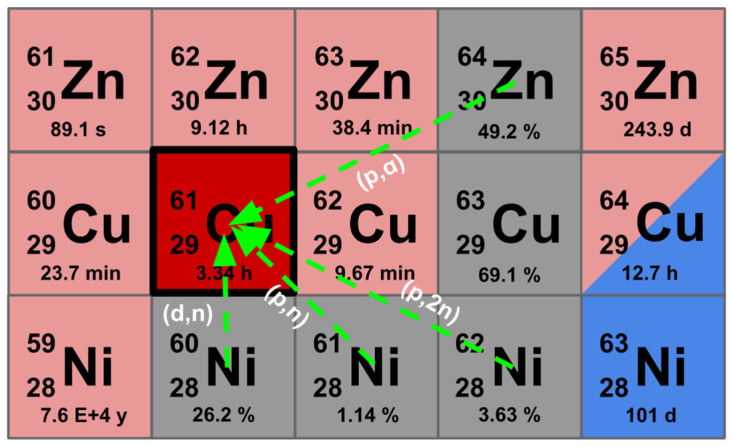
Main production routes for ^61^Cu are summarized, including target materials and corresponding nuclear reactions.

Clinical-scale production of ^61^Cu has been achieved using both nickel and zinc targets, in solid or liquid form. From nickel, the most widely studied pathway is the ^61^Ni(p,n)^61^Cu reaction, which offers high yields with cross sections of ~500 mb [46,51]. Some attention has also been given to the ^60^Ni(d,n)^61^Cu reaction (cross section ~200 mb) [46,52]. More recently, the ^62^Ni(p,2n)^61^Cu reaction has been investigated, with cross sections in the ~350 mb range [53]. While the (p,n) route on ^61^Ni provides the highest yields, its practical use is limited by the high cost of enriched target material, since ^61^Ni represents only ~1% of natural nickel. By contrast, ^62^Ni (3.6% natural abundance) is considerably less expensive, with enrichment costs at least threefold lower. Furthermore, the relatively high abundance of ^60^Ni (26%) allows for the use of natural nickel with deuteron irradiation as a viable alternative. Zinc targets have also been explored, particularly through the ^64^Zn(p,α)^61^Cu reaction. Although the peak cross section is more modest (~100 mb), the approach benefits from the high natural abundance of ^64^Zn (49%), enabling the use of both natural and enriched zinc [54,55,56]. Solid targets typically deliver higher yields owing to their greater nuclear density, whereas liquid targets offer the advantage of simplified handling and faster post-irradiation processing [57,58]. Starting either from nickel or zinc targets, activities in the multi-GBq range have been reported, confirming the feasibility of clinical production. The discussed nuclear reactions and target materials for ^61^Cu production are schematically summarized in Figure 2.

With its physical half-life and favorable dosimetry, ^61^Cu is particularly suited for labeling small molecules and peptides, where rapid pharmacokinetics require a radionuclide with compatible decay properties. Importantly, its combination of high-quality PET imaging characteristics and viable large-scale production routes supports its potential to complement—and in certain contexts, replace—currently established short-lived positron emitters such as ^68^Ga [27,38,59,60].

#### 2.1.3. Copper-64

Over the years, numerous nuclear reactions have been explored for the production of ^64^Cu. Among these, the ^64^Ni(p,n)^64^Cu reaction has emerged as the most practical and widely adopted approach, offering high yields of no-carrier-added ^64^Cu and robust reproducibility across multiple groups worldwide [51,57,61,62,63,64,65]. Comparable yields can also be achieved via the ^64^Ni(d,2n)^64^Cu reaction, although this pathway is less accessible due to the limited availability of deuteron accelerators [66]. The main drawback of the nickel-based routes is the high cost of the enriched target material [41,42]. Since ^64^Ni accounts only for ~0.9% of natural nickel, the highly enriched material required has a current price around 50 USD per milligram. Despite this economic barrier, the method remains the global standard, owing to its exceptionally favorable nuclear cross sections (approaching 1000 mb at optimal proton energies) and the availability of mature recycling protocols. Established target recovery methods routinely achieve efficiencies exceeding 90%, effectively amortizing the initial investment across multiple production cycles and making large-scale, routine supply feasible [57,67,68,69].

Other proton-based production alternatives feature the ^65^Cu(p,pn)^64^Cu and ^68^Zn(p,nα)^64^Cu nuclear reactions. The former offers an appealing cross section but suffers from intrinsically low molar activity, limiting its radiopharmaceutical utility [41]. The latter avoids the molar activity issue but delivers yields approximately an order of magnitude lower than those achieved with the nickel-based routes [42,70]. The ^64^Zn(p,2p)^64^Cu reaction has also been tested in the past, though with rather low yields [71].

Alternative neutron-based strategies have also been pursued. Production with thermal neutrons via ^63^Cu(n,γ)^64^Cu generally suffers from low specific activity due to the low reaction yield and the presence of bulk stable copper [43,72]. On the other hand, the use of fast neutrons through the ^64^Zn(n,p)^64^Cu reaction can, in principle, achieve molar activities comparable to cyclotron-produced ^64^Cu. However, the relatively low cross section of this reaction and the unavoidable co-production of long-lived ^65^Zn pose significant challenges [72,73,74].

Recently, accelerator-driven neutron sources have rekindled interest in neutron-based ^64^Cu production. In this emerging approach, high-energy protons or deuterons are directed onto a converter (typically beryllium or carbon) to generate secondary neutrons, or deuterons are fused with tritium to produce fusion neutrons. These secondary neutrons can then be harnessed for radiocopper production [75,76,77,78]. Such methods hold promise as complementary technologies to conventional cyclotron-based routes, particularly in facilities equipped with versatile high-current accelerators.

**Figure 3:**
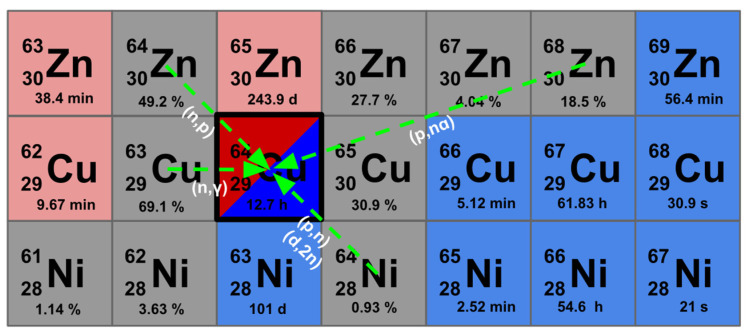
Main production routes for ^64^Cu are summarized, including target materials and corresponding nuclear reactions.

The discussed nuclear reactions and target materials for ^64^Cu production are schematically summarized in Figure 3.

While ^64^Cu has occasionally been considered for therapeutic purposes, its relatively short half-life (12.7 h) and the presence of a β^+^ emission component limits its attractiveness compared to its therapeutic analog, ^67^Cu [11,17,64]. Nevertheless, ^64^Cu maintains a relevant role in therapeutic contexts, since it frequently appears as a co-produced radionuclide during ^67^Cu production. This aspect will be further elaborated in the following section.

#### 2.1.4. Copper-67

The therapeutic counterpart, ^67^Cu, has long been considered a “difficult catch” in radionuclide production. In contrast to the diagnostic copper radioisotopes, all investigated production routes for ^67^Cu exhibit relatively modest nuclear cross sections, with peak values typically in the 10–30 mb range [15].

Early investigations explored nickel targets for the ^64^Ni(α,p)^67^Cu nuclear reaction [79,80,81]. However, this pathway has remained limited in practice due to two main constraints: the low natural abundance and high cost of enriched ^64^Ni, and the restricted availability of accelerators capable of delivering sufficiently intense α-particle beams.

Zinc targets have therefore attracted greater attention. The ^68^Zn(p,2p)^67^Cu reaction was studied in the past, but its clinical translation was hindered by significant challenges: (i) the unavoidable co-production of isotopic impurities, such as ^64^Cu and ^65^Cu via (p,nα) and (p,α) side reactions, and (ii) the limited availability of high-energy proton accelerators required to drive this nuclear reaction efficiently [82,83,84,85].

In recent years, the ^70^Zn(p,α)^67^Cu pathway has regained prominence. Although first proposed decades ago [86,87], it is only in the last few years that groups have demonstrated meaningful yields, reporting ^67^Cu activities in the 0.1–1 GBq range from compact cyclotrons operating below 30 MeV [65,88,89,90]. The central question now is whether compact cyclotrons—already widespread in the medical isotope production landscape—can deliver clinically relevant levels of ^67^Cu via this reaction.

Here the strictly low-energy ^70^Zn(p,α)^67^Cu nuclear reaction (less than 30 MeV) should be differentiated from the high energetic ^70^Zn(p,X)^67^Cu production pathway [91,92]. The former can be readily performed on compact medical cyclotrons, whereas the latter offers the prospect of higher yields but requires larger accelerators comparable to those used for the ^68^Zn(p,2p)^67^Cu reaction. However, the use of higher proton energies inevitably increases the occurrence of undesired side-reactions, which in turn generate (radionuclidic) impurities. As a consequence, achieving sufficient radionuclidic purity and molar activity remains a major challenge. Co-production of radioactive copper isotopes such as ^61^Cu and ^64^Cu, as well as stable ^63/65^Cu, is unavoidable. These impurities may originate from high-energy nuclear reactions on ^70^Zn itself or from residual isotopic contaminants (e.g., ^68^Zn) in the enriched target material. To mitigate these issues and improve yields, innovative strategies have been proposed, including layered ^70^Zn/^68^Zn targets that simultaneously exploit the ^70^Zn(p,X)^67^Cu and ^68^Zn(p,2p)^67^Cu nuclear reactions [93].

A related approach, the ^70^Zn(d,nα)^67^Cu reaction, has also been investigated [94,95]. While it offers the prospect of nearly doubling the yield compared to proton irradiation, it poses an even greater technical challenge in terms of target cooling, owing to the shallower penetration depth of deuterons relative to protons and the associated localized energy deposition.

Whether using proton- or deuteron-based nuclear reactions, relatively thin targets are required, and a variety of fabrication strategies have been explored to improve the inherently modest yields. Approaches range from conventional methods, such as electroplated zinc and pressed ZnO targets, to more advanced techniques, including ZnO deposition by sputtering or plasma spark sintering [96,97,98].

Nuclear reaction pathways involving fast neutrons have also been explored, with particular attention to the ^67^Zn(n,p)^67^Cu reaction [73,76]. More recently, the ^68^Zn(n,np)^67^Cu route has been investigated, an approach that has become increasingly relevant in light of advances in accelerator-based neutron sources, as discussed in the previous section [75,77,99]. Despite these developments, however, no neutron-driven strategy has yet demonstrated activities comparable to those obtained via proton-induced reactions.

Finally, the ^68^Zn(γ,p)^67^Cu photonuclear route has emerged as an increasingly promising option [99,100]. Enabled by the advent of high-intensity electron accelerators and advances in target technology, this method has recently achieved commercial-scale supply of ^67^Cu in the 10–100 GBq range [101,102], positioning it as one of the most viable large-scale production strategies at present.

**Figure 4:**
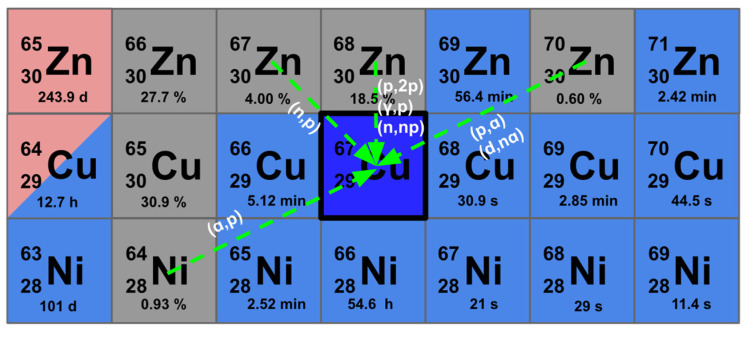
Main production routes for ^67^Cu are summarized, including target materials and corresponding nuclear reactions.

The discussed nuclear reactions and target materials for ^67^Cu production are schematically summarized in Figure 4.

### 2.2. Radiochemical Purification and Quality Control

#### 2.2.1. Radiochemical Purification

The radiochemical processing of irradiated targets for copper radioisotopes is typically performed using ion exchange or other chromatographic techniques, either as the sole separation step or in combination with additional methods. In charged particle-based production, relatively low target masses (50–200 mg) are employed, enabling separations through comparatively simple procedures. Typically, the target is dissolved in concentrated hydrochloric acid (6–10 M), followed by chromatographic separation using only a few columns [15,17]. As a general trend, the separation of copper from nickel targets is considered relatively straightforward, while zinc-based targets pose greater challenges. In the latter case, multi-step processes or intermediate neutralization steps are often required to achieve sufficient purity. These aspects have been comprehensively discussed in the recent work of Fonseca et al. [44].

In contrast, neutron- and photon-induced reactions generally require substantially larger target masses (5–100 g). This has prompted the development of bulk-separation approaches such as zinc sublimation to reduce the amount of non-radioactive material in solution [99]. A particular case is reactor-based ^64^Cu production via neutron capture, where chemical separation of the product from the bulk copper target is not feasible. Consequently, the product exhibits significantly lower molar activity compared to no-carrier-added methods employing nickel or zinc targets [72].

#### 2.2.2. Quality Control

Quality assessment of radiocopper solutions focuses on two main parameters: radionuclidic purity (RNP) and chemical purity. Radionuclidic purity is evaluated by gamma spectrometry. For short-lived isotopes such as ^60^Cu, ^61^Cu, ^62^Cu, and ^64^Cu, RNP values above 99% are routinely achieved at end of bombardment (EOB) [46,61]. For ^67^Cu, however, this value is often lower at EOB due to unavoidable co-production of shorter-lived copper radioisotopes [90,103]. Importantly, because of its longer half-life, the RNP of ^67^Cu improves over time as contaminants decay, with residual ^64^Cu being the most critical impurity.

To address this challenge, some groups have investigated the use of mixtures of ^64^Cu and ^67^Cu as a “theranostic cocktail”. Since achieving > 99% RNP would require impractically long cooling times after EOB, an alternative strategy has been to evaluate absorbed dose distributions. Modeling studies suggest that a mixture containing ~46% ^67^Cu and ~54% ^64^Cu results in a dose increment to healthy organs differing by less than 10% from that of pure ^67^Cu, underscoring its potential clinical value [104].

Chemical purity is assessed by measuring stable metallic impurities, most commonly originating from the target material (e.g., nickel, zinc) or from processing reagents and solvents. Reported impurities include iron, aluminum, and lead, in addition to stable copper, which may arise either from side reactions or environmental contamination [53,57,63].

The molar activity (MA) of a radiocopper preparation is defined as the radioactivity per mole of total copper (radioactive + stable). It is typically estimated from the measured activity and the concentration of stable copper in solution. Reported molar activities for radiocopper isotopes can reach up to 40% of the theoretical maximum, though in practice, higher values are difficult to achieve [63,69,88,103]. This limitation is largely due to the ubiquity of copper in the environment, which introduces numerous potential sources of contamination.

To better capture the functional performance of radiocopper preparations, the apparent molar activity (AMA) is often determined via titration with chelators such as NOTA, DOTA, or TETA [15,64,90]. Unlike MA, AMA reflects the influence of competing metal impurities that interfere with chelation and is, therefore, consistently lower. This distinction is particularly relevant for radiopharmaceutical applications and has been extensively studied by Søndergaard et al. for the case of ^67^Cu [105].

The most relevant production pathways for copper radioisotopes, along with reported yields and quality assessments, are summarized in Table 2, while detailed irradiation parameters can be found in the referenced literature.

So far, and to the best of our knowledge, only one manufacturer of radiocopper has been granted a marketing authorization by the European Medicines Agency (EMA) for ^64^Cu as radionuclide precursor [106,107]. It is noteworthy mentioning that the European Directorate for the Quality of Medicines & Healthcare (EDQM) is currently elaborating a monograph “Copper (^64^Cu) solution for radiolabeling” to define quality standards for ^64^Cu to be used in clinical applications of ^64^Cu-based radiopharmaceuticals [108]. Generally, automation of copper radiopharmaceuticals and their GMP compliance, as well as regulatory harmonization, will become more and more important for clinical translation [109,110,111,112,113,114,115].

**Table 2:** Summary of most relevant production pathways of copper radioisotopes with the reported yields and quality assessment. For details about irradiation parameters please see references. For the cross sections the recommended values from the IAEA Medical Portal were taken, and if not available the reference is shown in the table.

Radionuclide	Typical Production Route	Reported Peak Cross Section (Particle Energy)/mb (MeV)	Reported Activities	Reported RNP at EOB/%	Reported MA/AMA/GBq/µmol	Challenges for Clinical Translation
^60^Cu	^60^Ni(p,n)^60^Cu [46]	350 ± 100 [14,116]	>30 GBq	>99	>50 (AMA)	In-house production due to short half-life.
^61^Cu	^60^Ni(d,n)^61^Cu [46,52] ^61^Ni(p,n)^61^Cu [38,46] ^62^Ni(p,2n)^61^Cu [53] ^64^Zn(p,α)^61^Cu [54,59]	264 ± 13 [6] 470 ± 40 [11] 300 ± 150 [24,116] 71 ± 3 [16]	>2 GBq >20 GBq >20 GBq >3 GBq	>99 >99 >99 >99	>80 (MA) >50 (AMA) >130 (AMA) 1000 (AMA)	Mature production established.
^62^Cu	^62^Ni(p,n)^62^Cu [45] ^63^Cu(p,2n)^62^Zn → ^62^Cu [28,47]	530 ± 90 [12] 101 ± 5 [25]	XS measure >1.5 GBq	- >99	- -	Short half-life and short generator use.
^64^Cu	^64^Ni(p,n)^64^Cu [61,62,63,64] ^64^Ni(d,2n)^64^Cu [66,117] ^63^Cu(n,γ)^64^Cu [43] ^64^Zn(n,p)^64^Cu [74,118] ^68^Zn(p,nα)^64^Cu [42]	670 ± 50 [17] 960 ± 70 [21] ~10,000 (~keV) * [116] 250 ± 50 [11,116] 60 ± 4 [27]	>30 GBq >20 GBq >50 GBq >20 GBq ~5 GBq	>99 >99 >99 >99 >99	>1000 (AMA) >10 (AMA) ~0.2 (MA) >750 (AMA) >150 (AMA)	Mature production established. Neutron capture on copper leads to low MA.
^67^Cu	^64^Ni(α,p)^67^Cu [80] ^68^Zn(γ,p)^67^Cu [101] ^67^Zn(n,p)^67^Cu [73,119] ^68^Zn(n,np)^67^Cu [75,77] ^68^Zn(p,2p)^67^Cu [84] ^70^Zn(p,α)^67^Cu [90,103] ^70^Zn(d,nα)^67^Cu [95]	34 ± 4 [23,79] 11.7 ± 0.5 [23,116] 70 ± 40 [16,116] 110 ± 30 [26,116] ** 11.9 ± 0.9 [101] 12.4 ± 1.3 [16] 31 ± 1 [25,95]	~60 MBq >15 GBq ~1 GBq XS measure >10 GBq >1.5 GBq XS measure	- >99 - - <20 >98 -	- >120 (AMA) - - >1 (MA) >60 (AMA) -	Availability issues. So far, clinical amounts only by (γ,p).

* Cross section corresponding to resonances with epithermal neutrons. ** Simulated value, not empirically validated.

## 3. Preclinical and Clinical Work

The use of copper radionuclides in clinical applications has been summarized extensively [17,113,120,121,122,123,124,125,126,127]. The aim of this section is to highlight radiopharmaceuticals with potential for clinical routine demonstrated by initiation or completion of registered clinical trials or market approval (Table 3).

### 3.1. Copper-61

The use of ^61^Cu in clinical applications is scarce. Nevertheless, Fani and Nicolas describe the potential benefits of ^61^Cu as diagnostic radioisotope in future theranostic clinical studies [38] and Fonseca et al. demonstrated that ^61^Cu can even be produced in clinical amounts under GMP conditions which might also be advantageous over ^68^Ga in terms of logistics due to the longer half-life [59]. However, broader availability of ^61^Cu needs to be increased to be competitive to ^68^Ga. In that case, and if further positive outcomes of clinical trials are reported, ^61^Cu has the potential of significant growth in clinical use.

Besides a terminated study using [^61^Cu]Cu-ATSM [128], in a collaboration between the university hospitals of Basel and Munich (TUM) two first-in-human studies have been reported lately demonstrating the feasibility of clinical translation of ^61^Cu-radiopharmaceuticals from bench to bedside [26,27].

First, NODAGA-conjugated PSMA I&T was radiolabeled with ^61^Cu and evaluated in vitro and in vivo in LNCaP xenografts. Comparison against established PSMA-targeting radiopharmaceuticals like [^68^Ga]Ga-PSMA-11, [^68^Ga]Ga-PSMA I&T and [^18^F]PSMA-1007 revealed favorable data for [^61^Cu]Cu-NODAGA-PSMA I&T at late time points due to the longer half-life of ^61^Cu compared to ^68^Ga and ^18^F [26]. Although the limitation of this study was a single patient only, it resulted in the initiation and completion of a phase 1 clinical study with eight patients enrolled [129,130,131].

Second, a first-in-human application in the framework of the COPPER PET in NET study [132] comparing the SSTR antagonist [^61^Cu]Cu-NODAGA-LM3 head-to-head against the established SSTR agonist [^68^Ga]Ga-DOTA-TOC showing improved uptake in four matching liver lesions (SUV_max_ 25.4 vs. 23.2; SUV_mean_ of 12.2 vs.9.1) and a better tumor to background contrast (7.1 vs. 4.2) has been reported recently [27].

### 3.2. Copper-64

In recent years, ^64^Cu has been widely used clinically for targeting PSMA and SSTR where the feasibility has already been demonstrated with other radionuclides for either SPECT, PET or endoradiotherapy. Financial or logistical factors may support the wider use of ^64^Cu as radioisotope due to its longer half-life compared to ^68^Ga or ^18^F and the possibility to produce it in equal or larger amounts.

The somatostatin analog DOTA-TATE has been investigated for decades and has led to approval of ^177^Lu-radiolabeled Lutathera both in Europe and the US based on the results of the NETTER-1 trial [133,134,135]. [^64^Cu]Cu-DOTA-TATE has been applied at the Rigshospitalet Copenhagen first-in-human in 2012 [31] and since then successfully in more than 500 patients for the detection of neuroendocrine tumors [136]. [^64^Cu]Cu-DOTA-TATE has been approved by the FDA in 2020 as Detectnet after completing a prospective phase 3 trial [137,138]. In some studies using Lutathera, [^64^Cu]Cu-DOTA-TATE is now used as diagnostic companion for the treatment planning [139] and is the most used radiopharmaceutical labeled with ^64^Cu in clinical trials for different indications so far [137,139,140,141,142,143,144,145,146,147,148,149]. Comparable results have been shown by Hicks et al. using [^64^Cu]Cu-SAR-TATE in 10 patients with known neuroendocrine neoplasia at 30 min, 1h, 4 h, and 24 h after injection [150,151]. Further clinical studies with [^64^Cu]Cu-SAR-TATE have been completed [152,153,154].

The approval of [^177^Lu]Lu-PSMA-617, developed by the Heidelberg group [155,156,157], as Pluvicto in 2022 for the treatment of metastatic prostate cancer as one of the most common cancers lead to a renaissance of radionuclide theranostics in the last decade [158,159,160,161]. Numerous PSMA targeting radioligands have since then been developed [162,163]. Based on the same chemical strategy as [^64^Cu]Cu-SAR-TATE, [^64^Cu]Cu-SAR-bisPSMA has been evaluated by Zia et al. in LNCaP-tumor bearing mice showing high tumor uptake even after 24 h (SUV_max_ 12.9 ± 0.6, n = 3) [164]. Amongst completed early clinical trials [30,165,166,167], Gorin et al. reported initiation of a phase 3 clinical trial enrolling > 300 patients receiving 200 MBq of [^64^Cu]Cu-SAR-bisPSMA followed by same day (1–4 h p.i.) and next day (24 ± 6 h p.i.) PET imaging [168,169]. PSMA I&T, developed by the Munich group [170], has been radiolabeled with ^64^Cu and used in phase 3 clinical trials enrolling > 500 patients altogether [171,172] but no data has been published in the scientific literature yet. Radiolabeled bombesin analogs targeting the gastrin-releasing peptide receptor (GRPR) represent another class of radiopharmaceuticals suitable for radionuclide theranostics of prostate cancer in patients with negative PSMA expression [173]. Using [^64^Cu]Cu-SAR-bombesin [174] a detection rate of 44% was shown in biochemical recurrence prostate cancer patients with negative or equivocal PSMA PET/CT [175,176]. Besides prostate cancer, [^64^Cu]Cu-SAR-bombesin also shows potential for imaging estrogen receptor positive, progesterone receptor positive and human epidermal growth factor receptor 2 (HER2) negative metastatic breast cancer [177]. An antibody-based PSMA-targeting approach using [^64^Cu]Cu-TLX592 applying additional TLX592 in a dose escalation study has been reported as proof of concept for the planning of a prospective alpha therapy study using ^225^Ac [178,179].

Trastuzumab is a commonly used antibody to treat patients with breast cancer expressing HER2. For immunoPET imaging, ^89^Zr is often the radionuclide of choice as its physical half-life corresponds best with the biological half-life of the antibody [180,181,182]. From a dosimetry point of view, ^64^Cu has physical advantages over ^89^Zr. Mortimer et al. used [^64^Cu]Cu-DOTA-trastuzumab for immunoPET imaging of HER2 expression in 18 patients one day after injection [32]; however, the optimal time-point of imaging also depends on the technology, e.g., large field of view (LAFOV) PET scanners, being used [182]. The lack of a corresponding therapeutic radioisotope for ^89^Zr might be advantageous for copper radioisotopes, as shown by Rudd et al., who demonstrated efficacy of the theranostic pair [^64/67^Cu]Cu-Sar-trastuzumab for radioimmunotherapy in a preclinical setting of a HER2-expressing SKOV3 tumor model [183].

The City of Hope Medical Center completed a study where CD38, a transmembrane glycoprotein highly expressed on multiple myeloma cells, can be imaged with [^64^Cu]Cu-Daratumumab with improved specificity and sensitivity compared to [^18^F]FDG in 12 patients [184,185]. Integrin very late antigen 4 (VLA4 or α4β1) is a target also expressed on malignant multiple myeloma, which can be addressed using [^64^Cu]Cu-LLP2A ([^64^Cu]Cu-CB-TE1A1P-LLP2A) [186]. Laforest et al. determined safety and dosimetry of [^64^Cu]Cu-LLP2A in three multiple myeloma patients and six healthy volunteers [187,188]. Further investigations are ongoing, also for other indications like sickle cell disease [189,190,191]. More recently, the potential of [^67^Cu]Cu-LLP2A for endoradiotherapy in solid tumors using mice with B16-F10 melanoma has been shown preclinically [192]. Also, at the City of Hope Medical Center, the radiolabeled humanized anti-carcinoembryonic antigen (CEA) monoclonal antibody [^64^Cu]Cu-M5A showed tumor response in locally advanced rectal cancer and medullary thyroid cancer (MTC) with a further study ongoing [193,194,195].

The rise in fibroblast activation protein inhibitor (FAPI) imaging has created a large footprint in the field of nuclear medicine since its first reported results in the last decade [196,197,198,199]. The novel FAP targeted tracer [^64^Cu]Cu-RTX-1363S (now [^64^Cu]Cu-LNTH-1363S) has been preclinically studied in a U-87 MG tumor bearing mouse model and in 6 healthy humans [200,201]. [^64^Cu]Cu-LNTH-1363S is used in phase 1/2 studies as imaging agent in metastatic sarcoma and gastrointestinal tract cancer and as companion diagnostic in a therapeutic study treating sarcoma [33,202]. Another FAP tracer (FAP-2286) radiolabeled with either ^68^Ga or ^64^Cu is used in a phase 1 trial enrolling more than 150 patients with solid tumors [203,204]. Two prospective early phase studies using [^64^Cu]Cu-FAPI-XT117 have been completed in China [205,206,207].

Nectin-4 is a type 1 transmembrane cell adhesion protein overexpressed in bladder tumors [208]. After FDA authorization of the antibody drug conjugate enfortumab vedotin targeting nectin-4 and its high responding rates, a clinical need for precise diagnosis and therapy of nectin-4 expressing tumors is identified [209,210,211,212]. AKY-1189 is a mini-protein targeting nectin-4 and has been radiolabeled with ^68^Ga prior endoradiotherapy using ^177^Lu [210]. In a phase 1 study, alpha therapy using [^225^Ac]Ac-AKY-1189 is currently investigated in metastatic solid tumors in a cohort of 150 patients using [^64^Cu]Cu-AKY-1189 as companion diagnostic [213]. Based on preliminary work on the bicyclic peptide BT8009 [214], Krönke et al. developed the suitable conjugate NECT-224 for radiolabeling with ^68^Ga and ^64^Cu to target tumors with nectin-4 expression and demonstrated suitability first-in-human [215].

Urokinase-type plasminogen activator receptor (uPAR) is a biomarker in different human cancers [216]. [^64^Cu]Cu-DOTA-AE-105 is a linear peptide-based antagonist with high affinity to uPAR [217]. In a phase 1 trial four patients with prostate cancer, three patients with breast cancer and three patients with bladder cancer were imaged with [^64^Cu]Cu-DOTA-AE-105 without side effects [218,219]. Low risk prostate cancer patients are under investigation in a multi-center phase 2 trial [220].

Based on promising results from preclinical data [221], a group from Boston in the US used a 17 nm cross-linked carboxymethyl dextran nanoparticle (Macrin) conjugated to NODAGA and radiolabeled with ^64^Cu for PET imaging of macrophages in a recently reported phase 1 trial [222,223]. Results in seven healthy volunteers and three patients demonstrated potential for further studies in macrophage-associated diseases like cancer and sarcoidosis [223].

Natriuretic peptide receptors (NPR) play a major role in atherosclerosis, a cardiovascular disease. The expression of NP clearance receptors can be targeted by C-type atrial natriuretic factor (CANF) conjugated to DOTA and radiolabeled with ^64^Cu [224]. CANF can be incorporated in different concentrations into polymeric nanoparticles (comb) with 25% CANF content giving the best results in targeted PET imaging [225]. Safety and biodistribution of [^64^Cu]Cu-25%CANF-Comb has been demonstrated first-in-human and is now being used in clinical PET/MRI studies including >100 patients [226,227,228,229]. Within the same group [^64^Cu]Cu-DOTA-ECL1i was investigated to image C-C chemokine receptor 2 (CCR2) in diseases like myocardial inflammation or lung inflammation [230,231,232,233,234,235]. Further studies are ongoing including head and neck cancer, atherosclerosis, and pancreatic ductal adenocarcinoma [236,237,238].

[^64^Cu]Cu-FBP8, a short cyclic peptide, is used for fibrin-targeted PET/MRI hybrid imaging of thrombosis [239,240]. Dosimetry and safety of [^64^Cu]Cu-FBP8 has been determined in eight healthy volunteers and is now being studied in >100 patients with pulmonary embolism, deep venous thrombosis, atrial fibrillation, COVID-19, cancer, and Alzheimer disease [241,242,243,244].

Super paramagnetic iron oxide nanoparticles (SPION) is a cell-tracking technique that can be adopted to dual ^64^Cu PET/CT & PET/MRI [245,246]. Ciltacabtagene autoleucel (cilta-cel) is a B-cell maturation antigen-directed CAR T-cell therapy. Dowling et al. loaded 30% cilta-cel onto SPION and radiolabeled with ^64^Cu to monitor myeloma patients (n = 10) who received 70% pure cilta-cel prior [246,247].

miR-10b is a microRNA playing a fundamental role in metastatic cancer [248]. miR-10b inhibiting oligonucleotides were delivered to metastatic tumor sites using iron oxide coated nanoparticles (TTX-MC138) radiolabeled with ^64^Cu. In a phase 1 study feasibility of delivery of [^64^Cu]Cu-TTX-MC138 to tumor metastases was shown [249,250].

### 3.3. Copper-67

Clinical work using ^67^Cu has been reported decades ago [251]. However, registered clinical trials that have been executed so far are based on the work of the Donnelly group from Australia using a bifunctional sarcophagine (SAR—3,6,10,13,16,19-hexaazabicyclo(6,6,6)icosane) chelator to complexate ^67^Cu [252,253].

Paterson et al. conjugated Tyr^3^-octretotate to 5-(8-methyl-3,6,10,13,16,19-hexaaza-bicyclo [6.6.6]icosan-1-ylamino)-5-oxopentanoic acid (MeCOSar) to be used as radiocopper labeled SAR-TATE [150]. Feasibility of [^67^Cu]Cu-SAR-TATE was demonstrated preclinically by Cullinane et al. and Dearling et al. in ARJ42 xenografts and in a SSTR2-positive neuroblastoma tumor mouse model respectively [254,255]. Safety and efficacy of [^67^Cu]Cu-SAR-TATE have been evaluated in a phase 1/2 study in pediatric patients [256]. In another pilot study three patients with unresectable multifocal meningioma received four cycles of [^67^Cu]Cu-SAR-TATE after PET imaging with [^64^Cu]Cu-SAR-TATE [35]. A phase 1/2 trial using the theranostic pair [^64/67^Cu]Cu-SAR-TATE has been started [152]. In a preclinical study Ullrich et al. showed that including an albumin binder into the chemical structure of radioligands with SSTR2 affinity improves uptake and residence time in tumors and leads to equivalent efficacy in treatment as [^177^Lu]Lu-DOTA-TATE demonstrating the therapeutic potential of ^67^Cu as β^−^ emitter [257].

Preliminary preclinical results from Zia et al. and McInnes et al. using [^64/67^Cu]Cu-SAR-bisPSMA in PSMA-positive xenografts [164,258] showed feasibility for prostate cancer theranostics and initiated an ongoing phase 1/2 trial [34,259].

A preclinical therapy study with [^67^Cu]Cu-SAR-Bombesin was conducted in a gastric-releasing peptide receptor (GRPR) targeting PC3 tumor mouse model [260]. Potential for clinical translation of [^67^Cu]Cu-SAR-Bombesin is being evaluated in a phase 1/2 study [261].

**Table 3:** Summary of radiopharmaceuticals labeled with ^61^Cu, ^64^Cu or ^67^Cu and used in registered clinical trials.

Radiopharmaceutical	Radionuclide	Indication	Trial Acronym	Trial Phase	Enrollment	Trial Identifier	Status *	Reference
[^61^Cu]Cu-NODAGA-LM3	^61^Cu	Neuroendocrine tumors	Copper PET in NET	1/2	27	NCT06455358	Recruiting	[132]
[^64^Cu]Cu-SAR-bisPSMA	^64^Cu	Prostate cancer	Propeller	1	30	NCT04839367	Completed	[165]
[^64^Cu]Cu-SAR-bisPSMA	^64^Cu	Prostate cancer	Cobra	1/2	52	NCT05249127	Completed	[30]
[^64^Cu]Cu-SAR-bisPSMA	^64^Cu	Prostate cancer	-	1/2	150	NCT05286840	Unknown	[262]
[^64^Cu]Cu-SAR-bisPSMA	^64^Cu	Prostate cancer	Co-PSMA	2	50	NCT06907641	Recruiting	[263]
[^64^Cu]Cu-SAR-bisPSMA	^64^Cu	Prostate cancer	Clarify	3	383	NCT06056830	Recruiting	[168]
[^64^Cu]Cu-SAR-bisPSMA	^64^Cu	Prostate cancer	Amplify	3	220	NCT06970847	Recruiting	[264]
[^64^Cu]Cu-SAR-bisPSMA [^67^Cu]Cu-SAR-bisPSMA	^64^Cu ^67^Cu	Prostate cancer	Secure	1/2a	54	NCT04868604	Recruiting	[259]
[^61^Cu]Cu-PSMA I&T	^61^Cu	Prostate cancer	-	1	8	NCT06736054	Recruiting	[129]
[^64^Cu]Cu-PSMA I&T	^64^Cu	Prostate cancer	-	2	24	NCT05653856	Completed	[265]
[^64^Cu]Cu-PSMA I&T	^64^Cu	Prostate cancer	Solar-Recur	3	235	NCT06235099	Completed	[171]
[^64^Cu]Cu-PSMA I&T	^64^Cu	Prostate cancer	Solar-Stage	3	323	NCT06235151	Recruiting	[172]
[^64^Cu]Cu-DOTA-TATE	^64^Cu	Neuroendocrine tumors	-	3	63	NCT03673943	Completed	[137]
[^64^Cu]Cu-DOTA-TATE	^64^Cu	Neuroendocrine tumors/prostate cancer	-	-	50	NCT05680675	Completed	[142]
[^64^Cu]Cu-DOTA-TATE	^64^Cu	Endocarditis	-	-	69	NCT05432427	Completed	[141]
[^64^Cu]Cu-DOTA-TATE	^64^Cu	Neuroendocrine tumors	-	Expanded access	-	NCT04334837	Approved for marketing	[140]
[^64^Cu]Cu-DOTA-TATE	^64^Cu	Neuroendocrine tumors	-	1	10	NCT06122610	Recruiting	[139]
[^64^Cu]Cu-DOTA-TATE	^64^Cu	Lyme Neuroborreliosis	DOTA-Lyme	-	50	NCT06392815	Recruiting	[147]
[^64^Cu]Cu-DOTA-TATE	^64^Cu	Neuroendocrine tumors	-	2	200	NCT05709171	Enrolling by invitation	[145]
[^64^Cu]Cu-DOTA-TATE	^64^Cu	Breast cancer	-	2	30	NCT05880394	Active, not recruiting	[143]
[^64^Cu]Cu-DOTA-TATE	^64^Cu	Neuroendocrine tumors	-	4	6	NCT06016855	Recruiting	[146]
[^64^Cu]Cu-DOTA-TATE	^64^Cu	Meningioma	-	4	20	NCT06377371	Recruiting	[148]
[^64^Cu]Cu-DOTA-TATE	^64^Cu	Cardiac Sarcoidosis	CuDosis	Observational	76	NCT06131112	Recruiting	[144]
[^64^Cu]Cu-DOTA-TATE	^64^Cu	Neuroendocrine tumors	-	Observational	30	NCT07195500	Recruiting	[149]
[^64^Cu]Cu-SAR-TATE	^64^Cu	Neuroendocrine tumors	-	1	10	NCT04440956	Completed	[153]
[^64^Cu]Cu-SAR-TATE [^67^Cu]Cu-SAR-TATE	^64^Cu ^67^Cu	Meningioma	-	1/2a	5	NCT03936426	Completed	[152]
[^64^Cu]Cu-SAR-TATE	^64^Cu	Neuroendocrine tumors	Disco	2	45	NCT04438304	Completed	[154]
[^64^Cu]Cu-SAR-TATE [^67^Cu]Cu-SAR-TATE	^64^Cu ^67^Cu	Neuroblastoma	-	1/2	21	NCT04023331	Terminated	[256]
[^64^Cu]Cu-SAR-Bombesin	^64^Cu	Prostate cancer	BOP	2	30	NCT05613842	Completed	[175]
[^64^Cu]Cu-SAR-Bombesin	^64^Cu	Prostate cancer	Sabre	2	53	NCT05407311	Completed	[266]
[^64^Cu]Cu-SAR-Bombesin [^67^Cu]Cu-SAR-Bombesin	^64^Cu ^67^Cu	Prostate cancer	Combat	1/2a	4	NCT05633160	Terminated	[261]
[^64^Cu]Cu-TP3805	^64^Cu	Prostate cancer	-	1	25	NCT02989623	Completed	[267]
[^64^Cu]Cu-TP3805	^64^Cu	Prostate cancer	-	1	25	NCT02603965	Completed	[268]
[^64^Cu]Cu-TP3805	^64^Cu	Urothelial cancer	-	1	20	NCT03039413	Completed	[269]
[^64^Cu]Cu-TP3805	^64^Cu	Breast cancer	-	-	19	NCT02810873	Terminated	[270]
[^64^Cu]Cu-AKY-1189	^64^Cu	Nectin-4 positive cancers	Nectinium-2	1/2	150	NCT07020117	Recruiting	[213]
[^64^Cu]Cu-TLX592	^64^Cu	Prostate cancer	Cupid	1	14	NCT04726033	Completed	[178]
[^64^Cu]Cu-trastuzumab	^64^Cu	Breast cancer	Cu-64 HER2+	1	11	NCT00605397	Completed	[271]
[^64^Cu]Cu-DOTA-trastuzumab	^64^Cu	Breast cancer	-	-	18	NCT01093612	Active, not recruiting	[272]
[^64^Cu]Cu-DOTA-trastuzumab	^64^Cu	Breast cancer	-	-	10	NCT02226276	Active, not recruiting	[273]
[^64^Cu]Cu-DOTA-trastuzumab	^64^Cu	Breast cancer	-	2	18	NCT02827877	Active, not recruiting	[274]
[^64^Cu]Cu-DOTA-trastuzumab	^64^Cu	Breast cancer	-	4	10	NCT05376878	Recruiting	[275]
[^64^Cu]Cu-DOTA-trastuzumab	^64^Cu	Gastric cancer	-	-	8	NCT01939275	Completed	[276]
[^64^Cu]Cu-DOTA-daratumumab	^64^Cu	Multiple myeloma	-	1	12	NCT03311828	Completed	[184]
[^61^Cu]Cu-ATSM	^61^Cu	Tumor hypoxia	-	-	9	NCT04621435	Terminated	[128]
[^64^Cu]Cu-ATSM	^64^Cu	Rectum cancer	-	2	70	NCT03951337	Active, not recruiting	[277]
[^64^Cu]Cu-ATSM	^64^Cu	Cervical cancer	-	2	73	NCT00794339	Terminated	[278]
[^64^Cu]Cu-ATSM	^64^Cu	Tumor hypoxia	-	1	13	NCT04875871	Terminated	[279]
[^64^Cu]Cu-M5A	^64^Cu	CEA positive cancers	-	-	20	NCT02293954	Active, not recruiting	[193]
[^64^Cu]Cu-M5A	^64^Cu	CEA positive cancers	-	-	15	NCT05245786	Recruiting	[194]
[^64^Cu]Cu-LLP2A	^64^Cu	Lymphoma	-	1	42	NCT06636175	Recruiting	[189]
[^64^Cu]Cu-LLP2A	^64^Cu	Multiple Myeloma	-	1	10	NCT03804424	Terminated	[187]
[^64^Cu]Cu-LLP2A	^64^Cu	Sickle cell disease	-	1	20	NCT04925492	Recruiting	[191]
[^64^Cu]Cu-LNTH-1363S	^64^Cu	Sarcoma/Gastrointestinal tumors	Phantom	1/2	26	NCT06298916	Recruiting	[33]
[^64^Cu]Cu-LNTH-1363S	^64^Cu	Sarcoma	Atlas	1/2	26	NCT07156565	Recruiting	[202]
[^64^Cu]Cu-FAP-2286	^64^Cu	Solid tumors	-	1	191	NCT04621435	Recruiting	[203]
[^64^Cu]Cu-FAPI-XT117	^64^Cu	Malignant solid tumors	-	1	14	NCT05814835	Completed	[205]
[^64^Cu]Cu-FAPI-XT117	^64^Cu	Malignant solid tumors	-	1	15	NCT05930457	Completed	[206]
[^64^Cu]Cu-NODAGA-TTX-MC138	^64^Cu	Solid tumors	-	1	1	NCT05908773	Completed	[249]
[^64^Cu]Cu-DOTA-U3-1287	^64^Cu	Solid tumors	-	1	12	NCT01479023	Terminated	[280]
[^64^Cu]Cu-NOTA-EB-ss-CPT	^64^Cu	Colorectal cancer	-	1	10	NCT05891028	Unknown	[281]
[^64^Cu]Cu-GRIP B	^64^Cu	Genitourinary malignancies	-	1/2	91	NCT05888532	Recruiting	[282]
[^64^Cu]Cu-DOTA-AE105	^64^Cu	uPAR	-	1	10	NCT02139371	Completed	[218]
[^64^Cu]Cu-DOTA-AE105	^64^Cu	uPAR	uTRACE-101	2	168	NCT06474806	Recruiting	[220]
[^64^Cu]Cu-DOTA-alendronate	^64^Cu	Breast cancer	-	1	1	NCT03542695	Completed	[283]
[^64^Cu]Cu-DOTA-ECL1i	^64^Cu	Abdominal aortic aneurysm	-	1	50	NCT04586452	Completed	[232]
[^64^Cu]Cu-DOTA-ECL1i	^64^Cu	Head and neck cancer	-	1	11	NCT04217057	Terminated	[236]
[^64^Cu]Cu-DOTA-ECL1i	^64^Cu	Heart diseases	-	1	90	NCT05107596	Unknown	[230]
[^64^Cu]Cu-DOTA-ECL1i	^64^Cu	Atherosclerosis	-			NCT04537403	Recruiting	[237]
[^64^Cu]Cu-DOTA-ECL1i	^64^Cu	Lung inflammation	-	1	110	NCT03492762	Recruiting	[234]
[^64^Cu]Cu-DOTA-ECL1i	^64^Cu	Pancreatic ductal adenocarcinoma	-	1	69	NCT03851237	Active, not recruiting	[238]
[^64^Cu]Cu-25%CANF-Comb	^64^Cu	Atherosclerosis	Volunteer—BioDistribution and Safety Study	1	8	NCT02498379	Completed	[227]
[^64^Cu]Cu-25%CANF-Comb	^64^Cu	Atherosclerosis	-	1	44	NCT02417688	Completed	[228]
[^64^Cu]Cu-25%CANF-Comb	^64^Cu	Atherosclerosis	-	-	80	NCT05838547	Recruiting	[229]
[^64^Cu]Cu-FBP8	^64^Cu	Pulmonary embolus	-	1	80	NCT04022915	Recruiting	[243]
[^64^Cu]Cu-FBP8	^64^Cu	Thrombosis	-	1	165	NCT03830320	Recruiting	[241]
[^64^Cu]Cu-FBP8	^64^Cu	Alzheimer Disease	-	1/2	30	NCT05336695	Recruiting	[244]
[^64^Cu]Cu-Plerixafor	^64^Cu	CXCR4	-	1	2	NCT02069080	Terminated	[284]
[^64^Cu]Cu-Macrin	^64^Cu	Sarcoidosis, cardiovascular diseases, cancer	-	1	10	NCT04843891	Unknown	[222]
[^64^Cu]CuCl_2_	^64^Cu	Wilson disease	-	1	5	NCT07159581	Enrolling by invitation	[285]
[^64^Cu]Cu-Super paramagnetic iron oxide nanoparticle	^64^Cu	Extramedullary myeloma	Caramel	1	10	NCT05666700	Recruiting	[247]
[^64^Cu]Cu-NOTA-PSMAi-PEG-Cy5.5-C’	^64^Cu	Prostate cancer	-	1	16	NCT04167969	Recruiting	[286]
[^64^Cu]Cu-TRDC002	^64^Cu	Prostate cancer	-	1	6	NCT07236112	Not yet recruiting	[287]
[^64^Cu]Cu-Porphysomes	^64^Cu	Gynecological cancers	-	1	24	NCT06977126	Not yet recruiting	[288]

* Status checked on 4 December 2025.

## 4. Conclusions

The feasibility of *CopperNostics* has been demonstrated not only preclinically but also clinically in a number of registered clinical studies with one PET radiopharmaceutical already authorized in the US paving the way for further developments to address unmet clinical needs. Key bottlenecks identified include broader availability of radionuclides—in particular, with a focus on upscaling of ^67^Cu production—as well as regulatory harmonization. Relevant copper radioisotopes can now be produced in sufficient quantity and quality to produce radiopharmaceuticals for preclinical and clinical applications.

## Data Availability

No new data were created or analyzed in this study. Data sharing is not applicable to this article.

## Abbreviations

The following abbreviations are used in this manuscript:

AMA	Apparent molar activity
CANF	C-type atrial natriuretic factor
CAR	Chimeric Antigen Receptor
CT	Computer tomography
DOTA	2,2′,2′′,2′′′-(1,4,7,10-Tetraazacyclododecane-1,4,7,10-tetrayl)tetraacetic acid
EDQM	European Directorate for the Quality of Medicines & Healthcare
EMA	European Medicines Agency
EOB	End of bombardment
FAP	Fibroblast activation protein
FAPI	Fibroblast activation protein inhibitor
FDA	Food and drug administration
GMP	Good manufacturing practice
GRPR	Gastrin-releasing peptide receptor
HER2	Human epidermal growth factor receptor 2
MA	Molar activity
MRI	Magnetic resonance imaging
NODAGA	1,4,7-triazacyclononane,1-glutaric acid-4,7-acetic acid
NOTA	1,4,7-Triazacyclononane-1,4,7-triacetic acid
NPR	Natriuretic peptide receptor
PET	Positron emission tomography
PSMA	Prostate specific membrane antigen
RNP	Radionuclidic purity
SAR	3,6,10,13,16,19-hexaazabicyclo(6,6,6)icosane
SPECT	Single-photon emission computed tomography
SPION	Super paramagnetic iron oxide nanoparticles
SSTR	Somatostatin receptor
SUV	Standardized uptake value
TETA	1,4,8,11-Tetraazacyclotetradecane-1,4,8,11-tetraacetic acid
uPAR	Urokinase-type plasminogen activator receptor
